# Clinical utility of liquid biopsy for the diagnosis and monitoring of *EML4-ALK* NSCLC patients

**DOI:** 10.1515/almed-2019-0019

**Published:** 2020-03-18

**Authors:** Estela Sánchez-Herrero, Mariano Provencio, Atocha Romero

**Affiliations:** Molecular Oncology Laboratory, Biomedical Sciences Research Institute Puerta de Hierro Majadahonda University Hospital, Madrid, Spain; Medical Oncology Department, Puerta de Hierro Majadahonda University Hospital, C/ Manuel de Falla 1, Majadahonda, Madrid 28222, Spain

**Keywords:** anaplastic lymphoma kinase, circulating free DNA, exosomes, liquid biopsy, non-small-cell lung cancer, tyrosine kinase inhibitors

## Abstract

**Background:**

Genomic rearrangement in anaplastic lymphoma kinase (*ALK*) gene occurs in 3−7% of patients with non-small-cell lung cancer (NSCLC). The detection of this alteration is crucial as *ALK* positive NSCLC patients benefit from *ALK* inhibitors, which improve both the patient's quality of life and overall survival (OS) compared to traditional chemotherapy.

**Content:**

In routine clinical practice, *ALK* rearrangements are detected using tissue biopsy. Nevertheless, the availability of tumor tissue is compromised in NSCLC patients due to surgical complications or difficult access to the cancer lesion. In addition, DNA quality and heterogeneity may impair tumor biopsies testing. These limitations can be overcome by liquid biopsy, which refers to non-invasive approaches for tumor molecular profiling. In this paper we review currently available technology for non-invasive *ALK* testing, in NSCLC patients, based on the analysis of circulating tumor DNA (ctDNA), circulating tumor RNA (ctRNA), circulating tumor cells (CTCs), tumor-educated platelets (TEPs) and extracellular vesicles (EVs) such as exosomes.

**Summary and outlook:**

Non-invasive tumor molecular profiling is crucial to improve outcomes and quality of life of NSCLC patients whose tumors harbor a translocation involving *ALK* locus.

## Introduction

Lung cancer is the most commonly diagnosed cancer along with breast cancer, contributing to more than 8.8 million deaths every year [1]. Non-small-cell lung cancer (NSCLC), the most common subtype of lung cancer, is diagnosed in most cases in advanced stages, making not possible a curative treatment [2].

**Figure 1:**
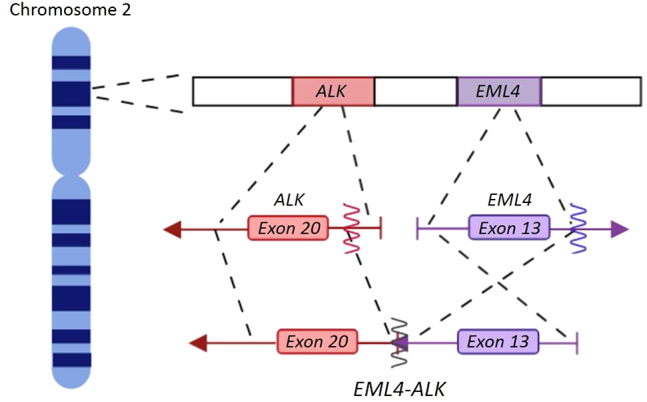
*EML4-ALK* translocation. *ALK* (red) and *EML4* (purple) genes are located in the short arm of chromosome 2 and they are oriented in opposite directions. Arrows indicate the orientation of the genes. The translocation occurs through a paracentric inversion [inv(2)(p21p23)] leading a fusion transcript that contains *ALK* catalytic domain and *EML4* amino-terminal half. (Figure designed by https://app.biorender.com/).

The anaplastic lymphoma kinase (*ALK*) gene, that was discovered in 1994 in anaplastic large cell lymphoma (ALCL), encodes a transmembrane receptor tyrosine kinase [3], whose alteration leads to constitutive *ALK* activation and generates oncogenic activity [4], [5]. Nearly 30 different *ALK*-fusion protein partners have been described and the most prevalent fusion partner in NSCLC patients is echinoderm microtubule-associated protein-like 4 (*EML4-ALK*) [6], [7] (Figure 1), leading to multiple fusions variants of which variant 1 (E13;A20, 33%), variant 2 (E20;A20, 10%), and variants 3 a/b (E6;A20, 29%) are the most frequent fusions [8].

*EML4-ALK* rearrangement occurs in 3−7% of NSCLC patients, which defines a specific molecular subtype of NSCLC [9], [10]. Interestingly, *ALK* rearrangement is observed predominantly in younger patients and never or light smokers with adenocarcinoma.

## *ALK*+ NSCLC treatment

**Figure 2:**
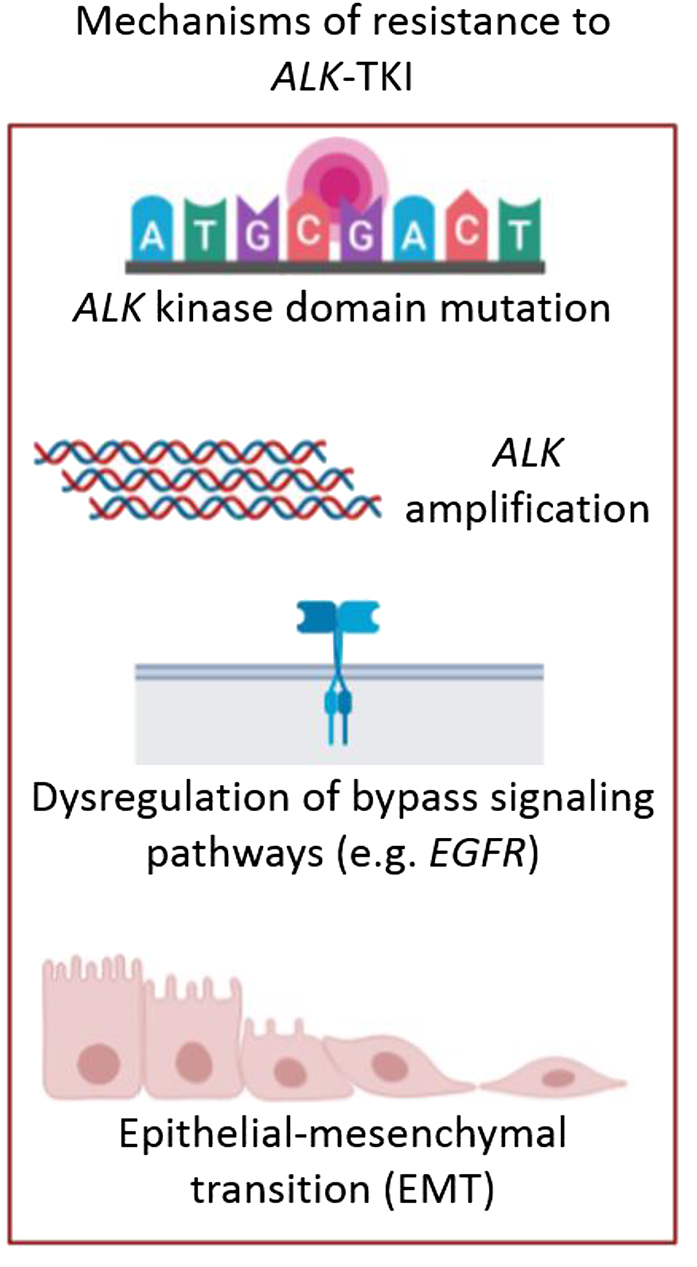
Treatment of *ALK*+ NSCLC patients. Mechanisms of resistance to *ALK*-TKIs: *ALK* kinase domain mutations (such as G1202R, G1269A, F1174L or L1196M), *ALK* amplification, dysregulation of bypass signalling pathways (such as EGFR, c-KIT, RAS-MAPK or PI3K-Akt activation) and epithelial-mesenchymal transition (EMT). (Figure designed by https://app.biorender.com/).

The development of tyrosine kinase inhibitors (TKIs) against *ALK*-gene has dramatically improved the patient's outcomes in terms of quality of life and prognosis, with significant higher progression-free survival (PFS), overall survival (OS) and objective response rates (ORRs) compared with chemotherapy [11], [12]. Thus, the identification of patients whose tumors harbor an *ALK* translocation is essential. Crizotinib, an ATP analog inhibitor of *ALK* that received approval from the US FDA (Food and Drug Administration) in 2011, is recommended as the first-line standard therapy for *EML4-ALK* advanced or metastatic NSCLC by the National Comprehensive Cancer Network (NCCN) guideline with an ORR of 74% and a PFS of 10.9 months [11]. However, despite its initial efficacy, resistance develops in approximately 73% of patients within 1–2 years of treatment [13]. Nevertheless, several second-generation *ALK* inhibitors have become available, such as ceritinib, alectinib and brigatinib, which received US FDA approval in 2014, 2015 and 2017, respectively. These therapies presented higher ORRs and PFS compared to platinum-pemetrexed based chemotherapy or crizotinib (ORRs of 73%, 83%, and 71%, and PFS of 16.6, 34.8 and not reported* months respectively [*median follow-up 11 months with brigatinib] [14], [15], [16]. Moreover, third-generation *ALK* inhibitors such as lorlatinib (a selective brain-penetrant *ALK* inhibitor) received FDA approval in 2018 [17]. Although next-generation *ALK* inhibitors are now available being more potent and effective than crizotinib against central nervous system metastases, little is known about the potential mechanisms of resistance to these agents. Several mechanisms of *ALK*-TKI resistance have been described (Figure 2), including *ALK* dependent mechanisms such as resistance mutations in the tyrosine kinase domain of *ALK* (G1202R, G1269A, F1174L or L1196M are the most frequent) and amplification of the *ALK* fusion gene, and *ALK*-independent mechanisms, as for example, dysregulation of bypass signalling pathways (*EGFR, c-KIT, RAS-MAPK, PI3K-Akt* activation, etc.) and epithelial-mesenchymal transition (EMT) [18].

However, the re-biopsy of the tumor is seldom performed in routine clinical practice in these patients, due to lung tumor difficult access. Thus, in most cases, *ALK*-TKIs resistance mechanisms are unknown, leading to an empirical prescription of subsequent lines of treatment, without knowing the tumor molecular profile upon disease progression. Noteworthy, it's known that *ALK* inhibitors have different binding affinities in the context of different resistance mutations; for example, early pre-clinical evidence suggests that the L1196M or S1206Y mutations confer resistance to crizotinib but not to ceritinib [19]. Thus, an optimal *ALK*-TKI sequence based on *ALK* mutations upon disease progression is necessary to allow clinicians to personalize *ALK*-targeted therapies, which will definitively improve the patient's outcome.

## Clinical practice in *EML4-ALK* patients

Different methods can be used to detect *ALK* rearrangements in tumor tissues. In routine clinical practice, the determination of *EML4-ALK* translocation is made by fluorescent *in situ* hybridization (FISH), immunohistochemistry (IHC) or next-generation sequence (NGS) [20]. Furthermore, it is important to highlight that when *EML4-ALK* translocation is identified by IHC and/or FISH, it is not possible to identify which variant is present. Although, all variants are oncogenic and induce *ALK* dependency [21], some studies have demonstrated that “short onco-proteins” resulting from *EML4-ALK* fusion variants such as variant 3 and variant 5 confer poorer outcome [22], [23]. Conversely, “long onco-protein” products of *EML4-ALK*, such as variant 2, are associated with better outcome [24]. Moreover, different *EML4-ALK* variants could have distinct sensitivities against the wide variety of *ALK*-TKI that are nowadays available. In this way, as in the case of *EGFR* mutations, in which tumors with exon 19 deletions are known to be more sensitive to TKIs than tumors harboring other alterations such as exon insertions in exon 20 [25].

In addition, methods such as immunocytochemistry (ICC), reverse transcriptase-polymerase chain reaction (RT-PCR), NGS and commercial kits are frequently employed. Finally, nCounter is a new technology capable of highly multiplexed analysis of different molecules such as RNA, miRNA, proteins and DNA. This methodology can detect *ALK* fusions using different starting material such as FFPE, fresh frozen tissues [16] or lung cancer patient-derived xenograft (PDX)-derived tumors [26].

Nevertheless, diagnosis based on tumor biopsies have some limitations such as the availability of samples for tumor molecular profiling, especially in lung cancer patients upon disease progression. Therefore, the lack of tissue biopsy condemns many patients with rearrangement in *ALK* to receive chemotherapy instead of *ALK*-TKIs, with a median overall survival (OS) of ∼12 months instead of ∼50 months from time of diagnosis of metastatic disease as reported by several observational studies using sequential *ALK* inhibitors [27], [28], [29], [30]. Moreover, the diagnosis based on the molecular profiling of a single biopsy may not reflect the overall situation of the entire tumor due to its heterogeneity [31]. Therefore, the development of methodologies that allow the non-invasive identification of *EML4-ALK* translocation, its variants and *ALK*-TKIs resistance mechanisms remains an unmet clinical need.

## Liquid biopsy

**Figure 3:**
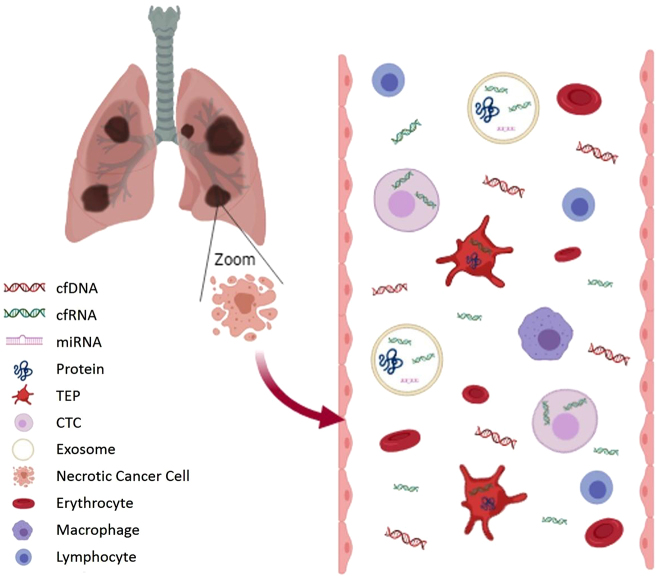
Components of liquid biopsy sample. cfDNA and cfRNA in blood come from dying cells (necrosis or apoptosis). A small fraction of cfDNA is ctDNA. Wild type DNA from blood cells can dilute ctDNA and therefore pre-analytical conditions should avoid cell lysis. CTCs, platelets (TEPs) and exosomes can capture these molecules and others, such as miRNA or cancer cell proteins. This genetic information is better protected in these compartments than in the bloodstream. (Figure designed by https://app.biorender.com/).

The term liquid biopsy refers to different methodological approaches including the study of circulating tumor DNA (ctDNA), circulating tumor RNA (ctRNA), circulating tumor cells (CTCs), tumor-educated platelets (TEPs) and extracellular vesicles (EVs; exosomes, microvesicles, microparticles, oncosomes) (Figure 3) [32].

Liquid biopsy, as a minimally invasive, safe and sensitive method, can overcome the aforementioned tissue biopsy limitations.

Tumor cells shed circulating tumor DNA (ctDNA) into the bloodstream, which can be subjected to molecular analysis. However, healthy cells also shed nucleic acids into bloodstream, as is the case of the erythrocytes, macrophages or lymphocytes. Therefore, it is important to extreme caution with pre-analytical conditions in order to avoid contamination of ctDNA with DNA that do not actually come from tumor cells (3). Importantly, the study of ctDNA requires methodologies with a very low limit of detection, 0.1% or lower, such as digital polymerase chain reaction (dPCR) [33], BEAMing [34], or NGS.

cfDNA profiling has proved to be useful to guide personalized treatments based on the molecular profile, for early detection of resistance mechanisms [35], [36], early detection of disease recurrences, as well as tumor response to therapy monitoring [37] by means of sequential liquid biopsies. Furthermore, several studies comparing the detection of *EML4-ALK* translocation by formalin-fixed paraffin-embedded (FFPE) tissues and liquid biopsy have reported that indeed cfDNA can be used as a surrogate of FFPE DNA [38].

Nevertheless, *ALK* fusion testing using liquid biopsies is rather challenging. First, genomic rearrangement identification using cfDNA is difficult as genomic brake-points are usually unknown and the rearrangement usually involves thousands of bp. Fusion transcripts although being more easy to detect requires to employ cfRNA which, unlike cfDNA, degrades very quickly due to the presence of RNases in the bloodstream. Therefore, it is plausible to hypothesize that a good strategy fusion testing would be the analysis of RNA that is contained in vesicles such as exosomes or platelets where it is protected from RNases.

## *EML4-ALK* detection strategies by liquid biopsy

### Circulating free DNA (cfDNA)

According to recent recommendations, liquid biopsy NGS panels using cfDNA is an adequate approach for the detection of *EML4-ALK* NSCLC patients resistance mutations when re-biopsy of the progression site is not feasible [19]. NGS seems to be the optimal method as it reaches levels of sensitivity and specificity around 80% of 100% respectively [39] and can provide information not only on resistance mutations of *ALK* gene but also on other molecular resistance mechanisms which may be target of a treatment either through a clinical trial or expanded access. However, the wide application of liquid NGS is currently limited owing to the need for specialized equipment and the high costs.

Conversely, alterations such as *EML4-ALK* translocation, can hardly be detected in the cfDNA since the brake-points at the DNA level is frequently unknown. Also, these alterations involve a large number of base pairs and cfDNA is fragmented (typically has a peak of approximately 150 bp). Thus, in contrast to *EGFR* mutations, *ALK* rearrangement detection from cfDNA is scarcely implemented in daily oncology.

Despite all of the above, *EML4-ALK* gene fusions have been detected by analyzing cfDNA, using a NGS liquid biopsy assay designed to detect the genomic breakpoint junctions. The assay was validated using custom cell lines with 10 *EML4-ALK* gene fusions and 26 synthetic fusions designed by computationally joining of the InVisionFirst Assay [40]. In addition, *ALK* fusion status has been analyzed in plasma cfDNA from NSCLC patients by using capture based NGS with a specificity of 100% [41], indicating that rearrangement detection through cfDNA sequencing is possible. However, due to the aforementioned limitations a negative result should be interpreted with caution.

### Circulating free RNA (cfRNA)

Complex aberrations such as large genomic rearrangements, including translocations, can be easily detected at the RNA level as fusion transcripts. Nevertheless, unlike cfDNA, free circulating RNA (cfRNA) degrades very quickly which constitutes an important limitation. Nonetheless, it has been reported that the optimization of the pre-analytical conditions of liquid biopsy samples can improve the sensitivity of RT-PCR based on cfRNA for the detection of *EML4-ALK* fusion transcripts [42].

Part of this cfRNA that circulates in the bloodstream is captured by diverse compartments, where it is more protected and functionally active [43]. Currently, the most frequently studied compartments include circulating tumor cells (CTCs), tumor-educated platelets (TEPs) and extracellular vesicles (EVs; exosomes, microvesicles, microparticles, oncosomes).

### Circulating tumor cells (CTCs)

Circulating tumor cells (CTCs) are viable circulating cells in the bloodstream of cancer patients that have been shed by a primary tumor. CTCs take part in metastasis as they are able to adhere to the wall of capillaries and penetrate a new tissue [44]. CTCs presence and counting has been reported to be associated with worse prognosis [45], [46]. Nevertheless, since CTCs are a minor fraction of cell population in the peripheral blood of cancer patients, both in terms of absolute (<10 cell/mL) and relative numbers compared to other blood cells (1 CTC per 106–107 leukocytes) [47], their use for the detection of *EML4-ALK* rearrangements must rely on highly efficient detection strategies. In addition, due to the heterogeneity of tumors, CTCs could present genomic variability, which implies a clonal heterogeneity of CTCs that must be taken into account [48]. Moreover, CTCs may present differences respect to primary and metastatic tumors [49].

Besides these limitations, *EML4-ALK* rearrangement has been detected in CTCs from peripheral blood of NSCLC cancer patients [45], [46], [47]. A variety of methods have been developed to isolate and identify CTCs; however, currently the only FDA-approved method for enumerating CTCs in blood samples is the CELLSEARCH® system [44]. Other methods used to detect *ALK* rearrangements rely on an initial enrichment process followed by CTCs detection, or vice versa, to increase the sensitivity and specificity [45].

Size of Epithelial Tumor Cells (ISET) technology is another method to isolate CTCs which has demonstrated greater effectiveness with respect to CELLSEARCH® system [50]. Using ISET followed by FISH and IHC, CTCs can be a reliable source for the detection of *ALK*-gene rearrangements in lung cancer patients, with ≥90% concordance with the tissue biopsy [51]. In addition, resistance mutations in *ALK* gene, such as L1196M, have been detected in CTCs [52] and can be expanded ex vivo for drug testing. Therefore, this source has clinical utility not only for diagnosis, but also a potential tool for drug sensitivity testing and for personalized precision medicine.

### Tumor-educated platelets (TEPs)

Platelets are cell fragments without nucleus originating from megakaryocytes in the bone marrow. These blood elements can sequester tumor related RNA by a microvesicle dependent mechanism, resulting in a change of its RNA and protein content [53]. As a result, these tumor-educated platelets (TEPs) have an altered function and can promote tumor cell survival and metastasis [53]. Furthermore, new evidence suggest that platelets are implicated in the immune responses and inflammatory diseases of the lungs [54]. Besides platelet content, count and size of platelets and platelet protein markers, such as Pselectin, are used for cancer diagnostics and prognostics [55]. However, platelets study has limitations such as a reducing count or potential activation due to certain therapies, which may affect the interpretation of the results.

*EML4-ALK* rearrangement has been identified by the analysis of RNA platelets of NSCLC patients before starting treatment with an *ALK*-TKI and upon disease progression, reappearing even before positron emission tomography- computed tomography (PET-CT) will show disease progression [56]. On the other hand, when patients respond to the treatment, *EML4-ALK* translocation was not detected in platelets. The sensitivity and specificity for the detection of *EML4-ALK* rearrangements in RNA isolated from platelets in patients has been reported to be between 65% and 100% respectively [56]. Even with the optimization of the pre-analytical conditions of liquid biopsy samples, the sensitivity of the detection of *EML4-ALK* rearrangements using cfRNA was lower compared to platelet RNA (21% and 65% respectively) [36].

Platelets can also capture extracellular vesicles (EVs), released by cancer cells, harboring tumor-specific RNA [53] such as tumor-derived *EML4-ALK* rearranged RNA. EVs are another starting material for nucleic acid isolation which are used for the study of *EML4-ALK* translocation by liquid biopsy samples.

### Exosomes

Exosomes are a type of EVs and refers to nanovesicles (30–200 nm) released after fusion of multivesicular bodies with the plasma membrane at the end of the endocytic recycling path. Exosomes with significant changes in their composition, are released by cancer cells and are able to act as a vehicle for the exchange of genetic and protein material between cells, which causes modifications such as angiogenesis, the acquisition of therapeutic resistance, the formation of metastases and an increase in proliferation [57].

Many potential non-invasive biomarkers that have been studied by liquid biopsy are commonly located in exosomes [58], [59]. Exosomal biomarkers could achieve a higher diagnostic and prognostic efficiency than using cfDNA alone [60].

Exosomes have not only been isolated from blood, but also from other biological fluids such as urine. However, the EVs concentration in urine is lower than in blood [48]. Many methods of exosomes isolation have been reported, being ultracentrifugation and some commercial kits the most commonly used. However, the efficiency and purity of the exosomes obtained by both strategies are different; ultracentrifugation has less efficiency but provides high purity, while commercial kits have lower purity due to improved efficiency [61], [62]. On the other hand, ultracentrifugation is a tedious method that takes a long time, so the improvement of new technologies such as commercially available kits or NGS-based methods by capture instead of by amplification could increase the sensitivity of *ALK*-fusion detection [63], [64]. Moreover, these new technologies may be easier and faster, so they could be implemented in routine clinical practice.

Few studies have analyzed fusion genes in exosomes. However, *EML4-ALK* translocation has been detected by the analysis of exosomal RNA, isolated from plasma samples from NSCLC patients, with a specificity of 100% and a sensitivity of 64–70% regarding tissue analysis [63], [65].

## Future approaches

Several studies have shown an excellent specificity for *EML4-ALK* detection by the different starting materials for nucleic acid isolation available in liquid biopsy considering tissue biopsy as the gold standard. Sensitivity, however, is rather low and negative results should be regarded with caution [41], [45], [56], [63]. Therefore, the challenge now is to increase the sensitivity of liquid biopsy for the detection of *ALK* rearrangements, improving both the isolation and detection technologies in order to establish robust and reproducible protocols. PCR based methods, dPCR, BeAMing, nCounter and NGS methodologies reach higher levels of sensitivity in the detection of *ALK* rearrangements compared with other strategies, although there are still some limitations.

Finally, evaluating *EML4-ALK* detection by different methods using liquid biopsy approaches in prospective cohorts is needed in order to establish diagnostic accuracy of different methodologies.

## Conclusions

Liquid biopsy is a non-invasive method, which can improve *EML4-ALK* translocation and *ALK*-TKIs resistance mechanisms detection, which will significantly improve *ALK*+ NCSCL diagnosis and patients management, leading to a better prognosis and quality of life of *ALK*+ NSCLC patients.
